# Noninflammatory Joint Contractures Arising from Immobility: Animal Models to Future Treatments

**DOI:** 10.1155/2015/848290

**Published:** 2015-07-13

**Authors:** Kayleigh Wong, Guy Trudel, Odette Laneuville

**Affiliations:** ^1^Bone and Joint Research Laboratory, Faculty of Medicine, University of Ottawa, 451 Smyth Road, Ottawa, ON, Canada K1H 8M5; ^2^Department of Medicine, Bone and Joint Research Laboratory, The Ottawa Hospital Rehabilitation Centre, 505 Smyth Road, Ottawa, ON, Canada K1H 8M2; ^3^Department of Biology, Faculty of Science, University of Ottawa, 30 Marie Curie, Ottawa, ON, Canada K1N 6N5

## Abstract

Joint contractures, defined as the limitation in the passive range of motion of a mobile joint, can be classified as noninflammatory diseases of the musculoskeletal system. The pathophysiology is not well understood; limited information is available on causal factors, progression, the pathophysiology involved, and prediction of response to treatment. The clinical heterogeneity of joint contractures combined with the heterogeneous contribution of joint connective tissues to joint mobility presents challenges to the study of joint contractures. Furthermore, contractures are often a symptom of a wide variety of heterogeneous disorders that are in many cases multifactorial. Extended immobility has been identified as a causal factor and evidence is provided from both experimental and epidemiology studies. Of interest is the involvement of the joint capsule in the pathophysiology of joint contractures and lack of response to remobilization. While molecular pathways involved in the development of joint contractures are being investigated, current treatments focus on physiotherapy, which is ineffective on irreversible contractures. Future treatments may include early diagnosis and prevention.

## 1. Introduction: Definitions and Diagnosis

The term “joints contractures” is used to describe the loss of passive range of motion of diarthrodial joints, the most common and movable type of joint. A functional classification of joints allows to best appreciate the importance of joint contractures for the most movable joints, diarthrodial, to synarthroses which are joints characterized by very little to no movement. In comparison, amphiarthrodial joints permit only slight motion, such as movement between vertebrae, and synarthroses (immovable joints such as sutures that connect skull bones) are attached by solid connective tissue. Characteristic of mobile diarthrodial joints is the synovial capsule that surrounds the joint space between bones and secretes the synovial fluid present in the articular space [1]. Therefore, joints can be classified by their function in terms of mobility. This classification system is preferable to use since function (i.e., range of motion or ROM) is the parameter used to define joint contractures. Joint contractures affect the essential function of movable joints by limiting movement and mobility, resulting in a negative impact on essential daily activity and healthy life style [2].

The measure of the passive or active range of motion (ROM) of a joint with a contracture is key to assessing the importance of joint contractures. An outside force, such as a therapist or physiotherapy equipment, measures the passive ROM by moving the joint through its natural range with no active effort from the individual. A goniometer quantitatively measures the angular distance of the joint motion and the loss of ROM in a contracture is usually recorded through comparison with the contralateral joint or normative values [3]. Conventionally, a joint contracture is named according to the joint involved and the direction opposite the lack of range. For the knee, the natural ROM from full extension at 180° to full flexion at approximately 40° is about 140°. A loss of knee ROM in extension (i.e., inability to fully extend) is referred to as a knee flexion contracture, compared to a loss in knee natural flexion amplitude, which is referred to as a knee extension contracture. Joint contractures can occur at any joint of the body and with a range of severity. Both the joint affected and severity of the joint contracture will dictate the impact on the patient. Knee flexion contractures affect gait and ambulation, while elbow flexion contractures limit only some of the movements performed by the arms, that is, those requiring elbow flexion. Joint contractures respond very poorly to currently available treatments [4, 5], which mainly involve physical therapy. Partial reversibility of a knee flexion contracture will improve ambulation but patients will still require assistance to ambulate since lack of extension of the knee joint of as little as 5° results in a limp [5] and gait will not be normal. The chronic nature of joint contractures, poor response to therapy, and negative impact on mobility make the limitation in the range of motion of joints one of the highest concerns of patients with arthritis, secondary only to pain [6, 7]. The loss of ROM not only impacts the affected joint but also negatively impacts the patient's ability to move and perform independent tasks, leading to a loss of autonomy and leaving individuals permanently crippled or handicapped [8].

## 2. Etiology of Joint Contractures

Circumstances leading to joint contractures vary significantly and the causal factors are not well understood. Patients diagnosed with joint contractures can be divided in three arbitrary groups: multiple congenital contractures, contractures in association with chronic diseases or after trauma, and contractures resulting from prolonged immobility. Patients with congenital disorders have multiple joint contractures involving more than one limb that are usually nonprogressive. The condition, termed arthrogryposis, occurs in 1/3,000 live births [9]. Congenital diseases characterized by the presence of joint contractures are associated with abnormalities of many of the genes involved in the development of connective tissues [10]. In the second group, contractures after trauma include post-fracture and connective tissue injury [11, 12]. Contractures arising after trauma to the joint may involve inflammatory pathways [13, 14] and are outside the scope of this review article. Contractures can be progressive and associated with chronic conditions such as arthritic diseases (rheumatoid arthritis and osteoarthritis (OA)) [15–17], total knee arthroplasty (TKA) [15, 18, 19], spinal cord injury [19–21], severe burn [19], brain injury [19, 22, 23], stroke [19, 24], obstetrical brachial plexus injury [19], muscular dystrophies [19, 25, 26], and diabetes [27, 28]. Capsular changes, including synovial proliferation and fibrosis, have been reported in arthritic patients and likely contribute, in concert with pain-limited ROM, to the common occurrence of contractures in this population. End-stage OA knees are treated with TKA. Knee contractures, present either before or after surgery, are associated with reduced success of TKA [18]. In a cohort of 5622 patients who underwent TKA, the incidence of postoperative flexion contractures was 3.6% at 2 years [18]. Of the 22,545 knee replacements performed in Canada in 2009-2010, 6.2% (1400) of patients were imposed with revision surgery to correct for contractures [29]. In the third group of patients, contractures result when mobility is reduced, such as the case in bedridden patients in intensive care units (ICUs) and institutionalized elderly people [5, 19, 30, 31]. A more than one-third incidence of developing a joint contracture in a large joint was documented for ICU patients with a hospital stay longer than 2 weeks [32]. A follow up of 155 patients who developed joint contractures while in the ICU revealed higher mortality 3.3 years after discharge [32]. Joint contractures are prevalent in institutionalized elderly people with chronic disease and 75% had knee flexion contractures that significantly limited ambulation [31]. In agreement, a recent study estimated the prevalence of joint contractures in nursing home residents at 55% with significant functional and medical consequences [30]. Immobility is a factor in each of these three groups. Disorders that cause multiple congenital contractures are all associated with decreased in utero movement [10]. In some instances, pathologies of the connective tissues of the joint develop first and lead to a reduced mobility of the joint. Chronic conditions often inflict pain on patients, leading to self-imposed or prescribed immobility. For example, patients with advanced knee OA will reduce the use of the affected joint, and over time a knee flexion contracture could develop. A lack of mechanical stimulation is a common thread in the etiologies of joint contractures.

The pathophysiology of joint contractures is highly heterogeneous; both environmental factors (e.g., mechanical stimulation) and various connective tissues of the diarthrodial joints are potentially involved. When the initiating factor is prolonged immobility, the mechanical properties of the healthy connective tissues of the joint will be altered negatively [11]. Mechanical stimulation of diarthrodial joints is clearly necessary to maintain joint function and homeostasis of the connective tissues. While the initiating event leading to joint contractures, prolonged immobility, or pathology of the connective tissues can be established, subsequent events and progression towards pathology are neither understood nor well documented.

## 3. Joint Tissues Restricting Range of Motion

The type of tissue that is restricting the ROM of the joint has been used to classify contractures [2]. Tissues that may be involved in the loss of ROM of a joint include muscles, capsule, tendons, ligaments, cartilage, skin, and bone. There is often a combination of multiple types of tissues involved, and it is difficult to isolate the contribution of individual joint structures to the limitation of ROM. Joint contractures arising from myogenic tissues (e.g., muscle and fascia) can occur in patients with neurologic conditions where defective motor neurons cause muscle spasticity [23, 33]. In Dupuytren's disease, a fibrotic cord in the palmar fascia of the hand creates contractures of the fingers' joints [34]. Contractures can also be cutaneous with skin causing a limitation in ROM in burn or scleroderma patients [2]. Contractures can also be identified as arthrogenic. Bone growth in the form of osteophytes or injury such as intra-articular fractures is known to lead to contractures [2]. Damage to connective tissues, such as the case in cartilage with osteochondritis dissecans or tears in the meniscus, may cause joint contractures [2]. In immobility-induced contractures, the capsule surrounding the joint has been identified as contributing to the irreversible limitation in ROM via capsular shortening, adhesive capsulitis, and/or arthrofibrosis [2]. There can be more than one type of tissue involved in the development of a contracture; for example, immobility leads to both muscle atrophy and changes in the capsule which both contribute to loss in ROM. The heterogeneous nature of the disease leads to difficulty in determining how much each tissue is involved and the potential targets for treatment.

## 4. Animal Models to Study the Pathophysiology of Joint Contractures

Animal models of joint contractures support the causal role of the capsule tissue in limiting ROM. In a rat model of immobilization-induced knee flexion contracture, the capsular contribution was demonstrated by sectioning the posterior knee joint muscles and failure to restore the full ROM thereby attributing the limitation to the capsule [35]. When the knee joint is flexed, the relaxed posterior capsule adopts a folded configuration. Extension of the knee unfolds the posterior capsule, which then adopts a fully stretched configuration. The opposing synovial intimas of adjacent folds will glide on each other in the flexed position. Immobilization in flexion alters capsule homeostasis: opposing synovial folds become adherent, which decreases the length of the posterior capsule synovial intima [36]. Long-lasting folded posterior capsule shows histological evidence of adhesions between folds that become organized and fused effectively shortening the posterior capsule length. This structural reorganization of the posterior capsule prevents unfolding and resists knee extension. Immobilization causes further reorganization of the synovial layer: decrease in synoviocyte proliferation and in the amount of synovial fluid [36–38]. In addition, the capsule undergoes subsynovial changes including disordered alignment of collagen fibers, increased type I collagen, and buildup of advanced glycation end products [39]. These structural alterations of the posterior capsule can explain the articular limitation in knee extension with long-lasting contractures.

The rabbit model of posttraumatic knee contractures simulating stable intra-articular fractures and accompanied by a period of immobilization also supports the capsular contribution to joint contractures. The numbers of myofibroblasts, described as fibroblasts expressing the *α*-type of smooth muscle actin, and of mast cells are elevated in the joint capsule [14]. Joint capsules are dynamic tissues, stretching and relaxing as a consequence of joint movement and mechanical stimulation appears essential for maintaining capsule elasticity.

## 5. Cellular and Molecular Development of Joint Contractures

While contractures can be associated with an inflammatory response resulting from direct injury to the joint (tears, fractures, etc.), joint contractures also arise without the classical signs of inflammation. Over 400 different conditions have been classified as arthrogryposis multiplex congenita, described as multiple joint contractures at birth [10]. In general, etiologies of arthrogryposis lead to a decrease in fetal movement (fetal akinesia), and the earlier the onset, the more severe the contractures [9, 10]. Fetal akinesia is associated with build-up of connective tissue around the joint, muscle atrophy from disuse, or abnormal joint surfaces [10]. Neuromuscular diseases and abnormal formation of muscle or nerves cause muscle weakness and decreased fetal movement resulting in contractures [33]. Mutations in specific genes have been associated with arthrogryposis multiplex congenita [10]. The genes identified are part of myopathic pathways and neuropathic pathways and/or are involved in connective tissues [10].

Genetic changes during joint contracture formation in animal models have also been investigated. In a well-established rat model of immobilization-induced knee flexion contractures that do not directly injure the joint [35], changes in gene expression in the chondrocytes of articular cartilage have been identified. In immobilized cartilage, there were increases in prothrombin expression [40], mRNA levels of chitinase like-3 [41], and myeloid cell leukemia-1 transcript [42]. Protein levels of cyclooxygenases (PGHS-1 and PGHS-2) increased in articular cartilage and decreased in the synovial lining of immobilized joints [43]. Experiments with different inbred rat strains provided evidence of a genetic contribution to immobilization-induced contractures [17], indicating that there are intrinsic genetic factors that influence the development of contractures.

The pathology of the development of joint contractures from immobilization has been studied for many decades and has produced many divided results. Proliferation of intra-articular tissue [44–50] and synovial adhesions to the articular cartilage followed by degradation [44, 45, 47, 48, 51] have been described. These findings were incongruous to other reports describing neither pannus proliferation nor adhesion to the cartilage [52–54]. These diverging results may be attributed to the different species, joints, and methods of immobilization used, some of which include direct injury to the joint. Using the established rat model, a reduction in synovial intimal length after immobility suggested that adhesions of the synovial intima occurred in joint contractures rather than pannus proliferation [36].

Joint contractures caused by immobility have both myogenic and arthrogenic components. In the rat model, immobilization periods of less than two weeks caused contractures that were mostly due to muscular limitation, and contractures were reversible with spontaneous remobilization [55]. When immobilized for four or more weeks, articular structures contributed more to the limitation of ROM and the resulting contractures were irreversible [55]. The main arthrogenic component that limits the range of motion is the posterior capsule [35]. The capsule forms a sleeve around diarthrodial synovial joints and is made of dense, fibrous tissue and is composed mostly of collagen protein [1]. Multiple groups have associated joint contractures with disruptions in collagen synthesis, organization, and posttranslational modifications [13, 39, 46, 50, 54, 56]. As far back as 1966, a study in rats and dogs provided evidence for an increase in collagen synthesis in the joint after immobilization [46]. In the rat model, experiments have shown higher amounts of type I collagen levels and lower amounts of type III collagen in capsule cells of immobilized legs compared to sham-operated legs, suggesting that the contractures were caused by fibrosis [15]. In another rat model, there was a significant increase in reduced cross-links of collagen in the form of advanced glycation end products (AGEs) [39]. These posttranslational modifications are known to increase stiffness in connective tissue [39, 57]. The role of AGEs is highlighted by a prevalence of several rheumatologic conditions in patients with diabetes caused by an excess of AGEs from the increased availability of glucose [58]. Disorganization of collagen fibres in the posterior capsule compared to nonimmobilized joints and a decrease in glycosaminoglycans were also reported in immobilized capsule [39, 53, 56]. Glycosaminoglycans are long polysaccharide chains that retain water and their loss may allow further collagen crosslinking [56]. Other changes in the posterior capsule include fewer proliferating synoviocytes and a decrease in synovial intimal length [36]. Human posterior capsule samples obtained from OA patients undergoing TKA showed an increase in collagenous tissue and a decrease of synovial tissue in the contracture group compared to the noncontracture group [17]. Although these results were not statistically significant, they coincide with previous results of increased fibrosis, decreased synoviocyte proliferation, and shortened synovial length. A shortening of the posterior capsule combined with fibrosis may be contributing to irreversible knee flexion contractures. Gene expression changes in the posterior capsule of immobilized knee joints have also been studied. Genome-wide gene expression analysis of posterior capsule in patients with OA and contracture showed a decrease in casein mRNA and increases in chondroadherin, angiogenic inducer CYR61, and SRY-box 9, four genes that have been associated with tissue fibrosis [15].

Muscle spasticity and atrophy can be treated, but disuse causes changes in arthrogenic structures that are irreversible. Irreversible contractures caused by the capsule can be detected by a “firm end-point” when measuring passive range of motion, as opposed to a “spongy end-point” which identifies a contracture that would effectively respond to physical therapy treatments [5]. Current treatments mostly include physical therapy; however, most contractures are diagnosed only when they become chronic and are unresponsive to rehabilitation. For a chronic joint contracture, rehabilitation with physiotherapy is the most common treatment, including stretching, exercise, and static and dynamic bracing. Complications can arise from these treatments, with risk of breaking skin, bleeding, ulcer formation, joint dislocation, and pain [2]. Stretch is also largely ineffective for people with neurologic conditions such as stroke, spinal cord, brain injury, or cerebral palsy [59]. If the contracture is severe and is unresponsive to conventional treatments, joint capsule release surgery is an option [60]. Surgery can be effective, but it is technically demanding and risks creating instability in the joint or damage to critical neurovasculature [15, 61]. In general, current treatments are not effective and the disease permanently impairs the physical function of individuals. There are a few pharmacological treatments that have potential for increasing range of motion in joint contractures. Purified collagenase enzymes, currently approved for treatment of Dupuytren's hand contractures and Peyronie's penile contractures [62], have the potential to target the increased fibrosis of the posterior capsule. Intra-articular injections of decorin in a rabbit model have been shown to alter the expression of fibrotic genes [63] and may have the capability of reducing severity of joint contractures. Intra-articular hyaluronic acid injections with distension have improved pain and range of motion in patients with adhesive capsulitis of the shoulder [64]. Pharmacological interventions with intra-articular injections into the joint have the potential to be an effective mode for future treatment of joint contractures.

## 6. Conclusion

Prevention of the development of joint contractures would be the best course of action; however, contractures are often diagnosed when they are chronic and irreversible. Since contractures develop slowly overtime, it is difficult to identify in the preliminary stages [2]. Early diagnosis and identification of patients who will not respond to standard physiotherapy is key for effective and efficient treatment [65]. In patients with OA undergoing knee replacement surgery, preoperative reduced surgical knee flexion and reduced contralateral knee extension were associated with contractures [17]. Development of a joint contracture in the contralateral knee is mirrored in a rabbit knee flexion contracture model where a significant loss of range of motion was measured compared to an unoperated rabbit [66]. In patients, observation of decreased bilateral knee ROM could allow for early intervention. Prevention can be employed by identifying patients that are susceptible to joint contractures and ensuring stimulation of the joint through load bearing and range of motion exercises. When prevention is no longer an option, identifying patients that will respond well to physiotherapy compared to patients that will not respond (soft end-feel versus hard end-feel) would be an efficient use of resources. Those patients with contractures that would not respond to conventional treatments can explore other treatment options (surgery and pharmacological intervention) or, depending on severity, can be taught techniques that would facilitate an independent lifestyle with contracture. Prevention by prioritizing early diagnosis of patients with emerging joint contractures should be emphasized, since rehabilitation for less severe contractures is a more efficient and manageable process. Overall, joint contractures are a complex, heterogeneous, and multifactorial disease that, with contemporary treatments, is difficult to reverse.
